# 6-Halo-2-pyridone as an efficient organocatalyst for ester aminolysis†

**DOI:** 10.1039/d1ra04651a

**Published:** 2021-07-14

**Authors:** Takeshi Yamada, Yusuke Watanabe, Sentaro Okamoto

**Affiliations:** Department of Materials and Life Chemistry, Kanagawa University 3-27-1 Rokkakubashi, Kanagawa-ku Yokohama 221-8686 Japan tyamada@kanagawa-u.ac.jp okamos10@kanagawa-u.ac.jp

## Abstract

It was found that 6-halo-2-pyridones catalysed ester aminolysis in which not only reactive aryl esters but also relatively less reactive methyl and benzyl esters could be used as a substrate. The reaction could be performed without strictly dry and anaerobic conditions and the 6-chloro-2-pyridone catalyst could be recovered quantitatively after reaction. The method could be applied to dipeptide synthesis from methyl or benzyl esters of amino acids, where a high enantiomeric purity of the products was maintained. The mechanism involving dual activation of ester and amine substrates through hydrogen bonding between catalyst and substrates is proposed where 6-halo-2-pyridones act as a bifunctional Brønsted acid/base catalyst.

## Abstract 2

6-Halo-2-pyridones effectively catalyse ester aminolysis as bifunctional catalysts. This reaction did not require any special conditions and was operationally convenient.

## Graphical abstract

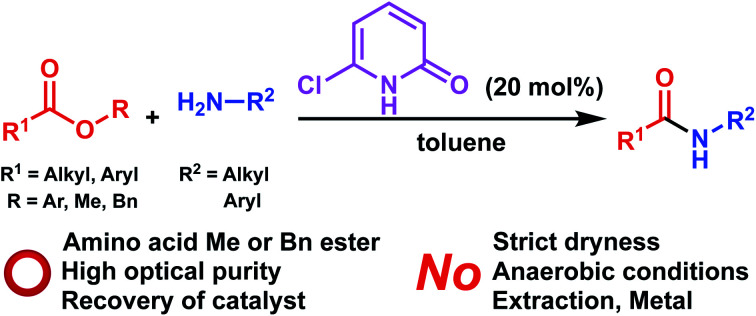

## Introduction

An amide bond is one of the most frequently observed chemical bonds in biologically relevant and functional molecules. The development of chemically and economically efficient methods for creating amide bonds is an ongoing research topic among organic chemists. Generally, amide bonds can be formed from carboxylic acids and amines using a stoichiometric condensation reagent.^1^ However, this promising method has economic and atom-economic drawbacks. Therefore, the formation of amide bonds from amines and carboxylic acids under catalytic conditions has become a recent trend in organic chemistry.^2^ Another method for creating an amide bond is the acylation of an amine with acid derivatives, such as acid anhydrides, halides and others.^3^ In addition, several amine equivalents such as isocyanide^4^ and azide^5^ were applied to form amide bonds. However, these methods require tedious preparation of substrates unless they are ordinary simple reagents.

The direct amidation of esters is also an effective method, particularly in the case of the elongation of a peptide from the C-terminus, and this is based on their availability from commercial sources and their ease of preparation. In the case of peptide elongation from the C-terminus, the ester was primarily hydrolysed prior to condensation with an amine in the presence of an appropriate stoichiometric amount of condensation reagent. Saponification of esters sometimes causes undesired reactions. Direct amidation of the ester avoids the need for a two-step process in this regard.

There are many reports on the direct amidation of esters with amines.^6–14^ Although many reactions are limited to relatively reactive phenyl esters or specific substrates, some reactions tolerate a broad range of substrates, such as the commonly used methyl or ethyl esters of amino acids, and provide the corresponding peptide without decreasing optical purity.^6*c*–*e*,8*e*,9*b*^ However, these reactions require strict dryness and anaerobic conditions. In addition, many reactions require expensive or commercially unavailable catalysts that cannot be reused.

**Scheme 1:**
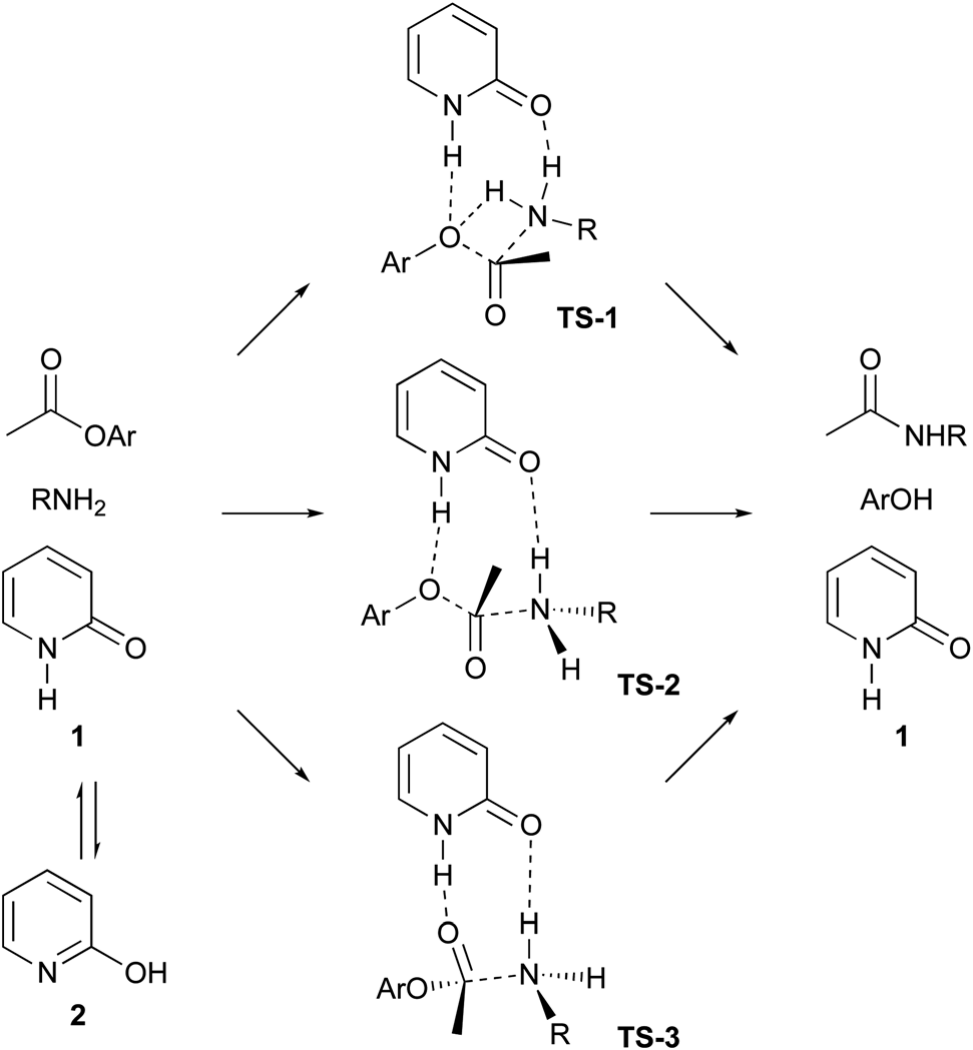
Proposed mechanism of 2-pyridine catalyzed ester aminolysis.

Ester aminolysis catalysed by neutral organomolecules such as N-heterocyclic carbenes,^12^ polyethers^13^ and 2-pyridones^14^ is an attractive reaction because of its mild reactivity and operational convenience. Ester aminolysis catalysed by 2-pyridone (1) was first reported by Rony and Openshaw's groups, independently.^14^ In this process, 2-pyridone (1) behaves as a bifunctional catalyst, the NH group activates an ester group to function as a Brønsted acid, and the CO group activates the amine as a Brønsted base.^14,15^ The proposed reaction mechanisms are described in Scheme 1, which shows three possible approaches. Path A involves four-membered ring transition state 1 (TS-1), and paths B and C include using eight-membered ring transition state 2 (TS-2) and 3 (TS-3). The NH group of 1 creates a hydrogen bond to aryloxygen to activate the ester in TS-2. The TS-3 approach includes the hydrogen bonding between NH group of 1 and ester carbonyl oxygen. A lactim tautomer, 2-hydroxy pyridine (2), can also be considered to function as a bifunctional catalyst.^15^ Although several mechanistic investigations have been conducted to date, only one study focused on the development of a highly reactive 2-pyridone type catalyst.^15*a*,*b*^ We are interested in the ability of 2-pyridone as an acid–base conjugate catalyst, and we reported on the organocatalytic α-addition of isocyanide with aldehyde using 3,5,6-trifluoro-2-pyridone as a catalyst.^16^ In this manuscript, we report on organocatalytic ester aminolysis using 6-halo-2-pyridones as a reactive catalyst.

## Results and discussion

Aminolysis of highly reactive *p*-nitrophenyl ester 3 with benzylamine (1.2 eq.) in the presence of 1 (20 mol%) in CDCl_3_ was examined (Table 1, entry 1). After stirring at room temperature for 5 h, 3 was converted to the corresponding amide 4 in 37%. The conversion was calculated by ^1^H NMR spectroscopy. The reaction without a catalyst achieved only 14% conversion (entry 2). Additionally, *N* or *O*-methylated 2-pyridone (6, 7a) revealed lower reactivity compared with 1 (entries 1, 3 and 4). These results support the proposed mechanism described in Scheme 1. Among the screened 2-amino or 2-mercapto-heteroarene catalysts 7b–d, 8 and 9, 2-pyridone (1) showed the highest reactivity (entries 1 and 5–12).^17^

**Table 1:** Catalyst screening

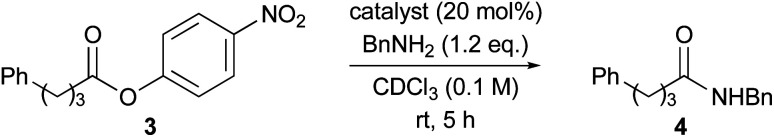		
Entry	Catalyst	Conversion^a^
1	2-Pyridone (1)	37%
2	None	14%
3	*N*-Methyl 2-pyridone (6)	25%
4	2-Methoxypyridine (7a)	17%
5	2-Mercaptopyridine (5)	30%
6	2-Aminopyridine (7b)	20%
7	2-Acetylaminopyridine (7c)	20%
8	2-Ethylaminopyridine (7d)	22%
9	2-Mercapto-1-methylimidazole (8)	32%
10	2-Mercaptobenzimidazole (9a)	22%
11	2-Mercaptobenzoxazole (9b)	30%
12	2-Mercaptobenzothiazole (9c)	31%
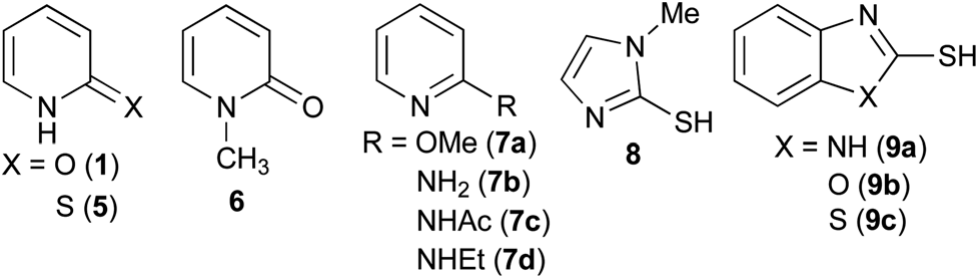		

a Conversion was calculated by ^1^H NMR spectroscopy.

**Fig. 1:**
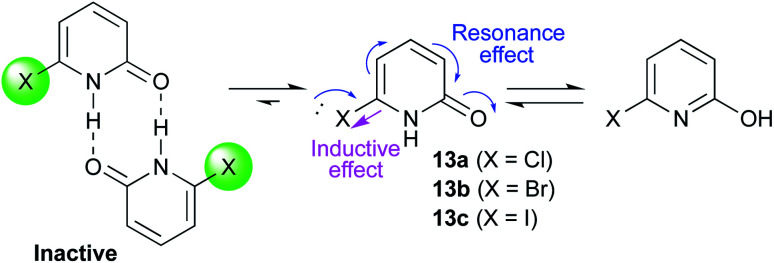
6-Halo-2-pyridone 13a, 13b and 13c.

A series of 2-pyridones, including electron-withdrawing and donating substituents at the C3, C4, C5 and C6 positions, were examined as potential catalysts. Selected examples are shown in Table 2. The introduction of electron-withdrawing and donating substituents at the C3 or C4 position did not improve catalytic activity (18–37% conversion, entries 1–6). Additionally, C5-substituted 2-pyridones 12 showed a similar catalytic activity to C4-substituted 2-pyridones 11, but 5-chloro-2-pyridone (12b) revealed slightly higher reactivity compared with 1 (entry 8). A substituent at the C6 position of 2-pyridone was found to be important for catalytic activity. A series of 6-substituted 2-pyridones 13 were examined. Among them, 6-halo-2-pyridone (13a–c) showed the highest catalytic activity for ester aminolysis (entries 10–16). It was assumed that the introduction of electron-withdrawing halogens increased the acidity of the 2-pyridone NH group, which activated ester moiety (Fig. 1).

**Table 2:** Substituent effect of 2-pyridone catalyst

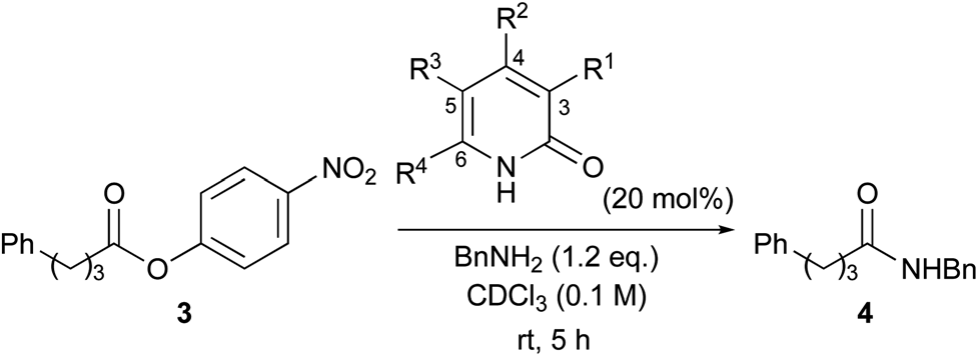			
Entry		R	Conversion^a^
1	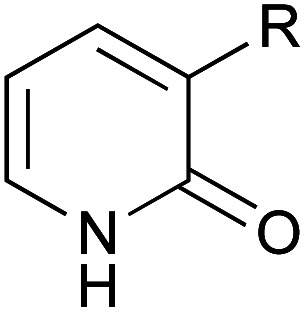	NO_2_ (10a)	23%
2		Cl (10b)	27%
3		OMe (10c)	34%
4	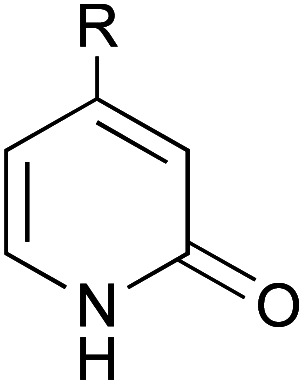	CF_3_ (11a)	32%
5		Cl (11b)	37%
6		OMe (11c)	34%
7	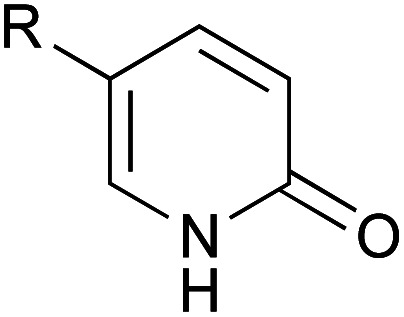	NO_2_ (12a)	42%
8		Cl (12b)	51%
9		OMe (12c)	41%
10	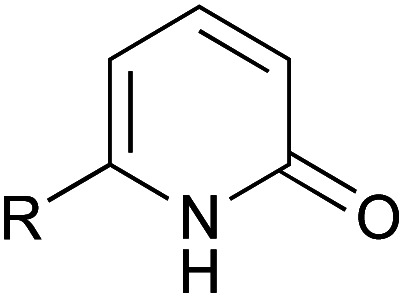	Cl (13a)	81%
11		Br (13b)	78%
12		I (13c)	86%
13		F (13d)	31%
14		CO_2_Me (13e)	50%
15		OMe (13f)	52%
16		CH_3_ (13g)	34%
17	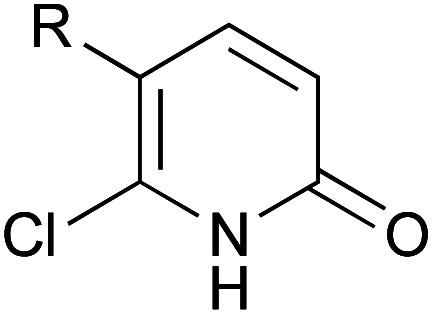	Cl (14a)	58%
18		NO_2_ (14b)	22%

a Conversion was calculated by ^1^H NMR spectroscopy.

The basicity of the carbonyl group of the catalyst may also have been increased by the resonance effect of halogens. The steric bulkiness of the C6 substituent may have inhibited the undesired homodimerisation of the catalyst.^15*a*^ In addition, halogen substituents improved solubility in organic solvents. A certain interaction of halogen atom to the substrate may assist this reaction. However, further introduction of electron-withdrawing groups to the C5 position decreased catalytic activity due to a decrease in the Lewis basicity of pyridone carbonyl (entries 17 and 18). Considering the availability and stability, further investigation was carried out using commercially available 6-chloro-2-pyridone 13a as a catalyst.^18,19^

Using 6-chloro-2-pyridone (13a) as a catalyst, various solvents were examined for this reaction. Selected examples are shown in Table 3.^17^ In this study, relatively less-reactive phenyl ester 15 was used as a substrate. The reactions were carried out in the presence of 20 mol% of 13a at room temperature for 5 h. The reaction proceeded at a 24% rate in CHCl_3_ (entry 1). The use of diethylether as a solvent showed almost the same results (entry 2). Aromatic solvents, particularly toluene, improved the conversion rate (entries 3–6). A non-polar solvent such as hexane exhibited the highest conversion rate of 74% (entry 7). Conversely, polar solvents such as DMF and *t*-BuOH were ineffective, and the rate was as low as that in the absence of a catalyst (entries 8 and 9). The reaction rate reduced dramatically without a catalyst in each solvent. These results clearly indicate that the hydrogen bonds between the catalyst 13a and the substrates are crucial for this ester aminolysis.

**Table 3:** Study of solvent effect

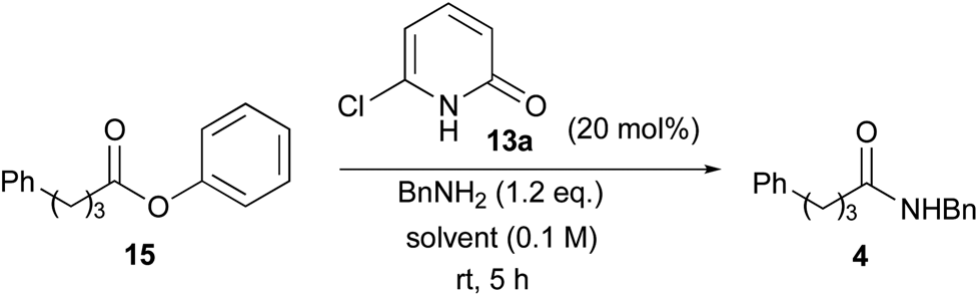			
Entry	Solvent	Conversion^a^	Conversion^a^ without catalyst
1	CHCl_3_	24%	—
2	Et_2_O	29%	2%
3	Benzene	46%	—
4	Toluene	52%	2%
5	Chlorobenzene	48%	—
6	CF_3_C_6_H_5_	48%	—
7	Hexane	74%	3%
8	DMF	13%	—
9	*t*-BuOH	18%	—

a Conversion was calculated by ^1^H NMR spectroscopy.

The reactions of a series of amines with phenyl ester 15 in the presence of 20 mol% of 13a were carried out in C_6_D_6_, the conversion of which was determined by ^1^H NMR analysis (Table 4). All reactions were conducted in 1.0 M solution due to the slow reaction in 0.1 M solution (entry 1). Triethylamine was included as an additive when using amine HCl–salt. Aminolysis of phenyl ester 15 with aromatic amine 16, branched amine 17 and secondary amines 18 and 19 was accelerated in the presence of catalyst 13a compared with catalyst-free conditions (entries 2–5). However, ester aminolysis with a highly hindered amine and methanolysis did not proceed at all (entries 6 and 7).

**Table 4:** Scope and limitation, focused on amine

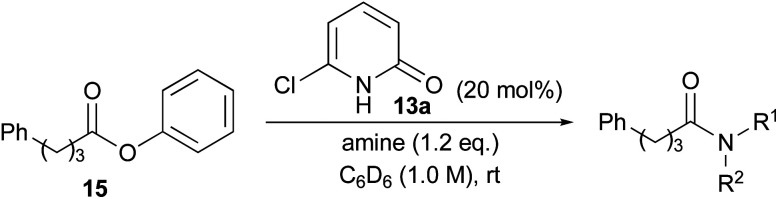				
Entry	Amine	Time	Conversion^a^	Conversion^a^ without catalyst
1^b^	Aniline (16)	32 h	2%	—
2	16	70 h	60%	Trace
3	1-Phenylethylamine (17)	49 h	87%	14%
4^c^	MeO(Me)NH·HCl (18)	48 h	73%	20%
5^c^	H-Pro-OBn·HCl (19)	47 h	68%	22%
6	*t*-BuNH_2_ (20)	50 h	n.r.^d^	n.r.^d^
7	MeOH	20 h	n.r.^d^	n.r.^d^

a Conversion was calculated by ^1^H NMR spectroscopy.

b Run in 0.1 M solution.

c 1.0 eq. of NEt_3_ was added.

d No reaction.

**Fig. 2:**
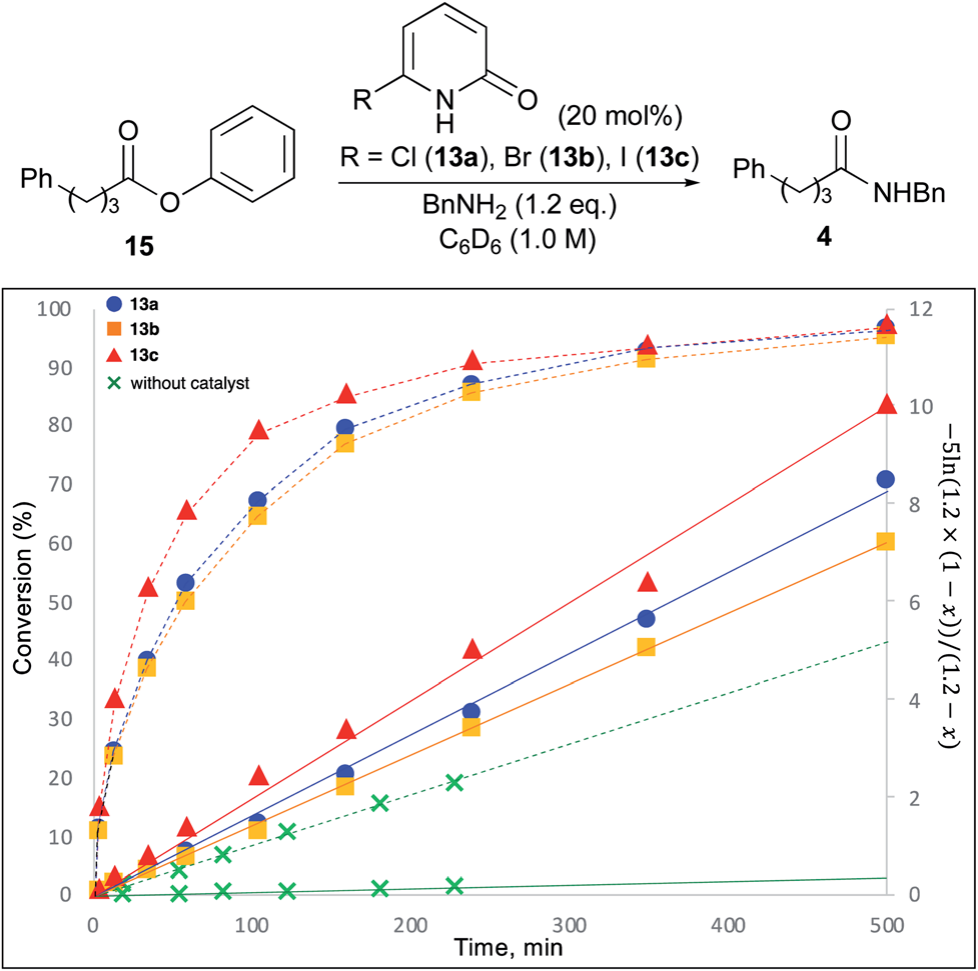
Kinetic study. Time course of conversion was plotted as a dotted line. Time *vs.* −5 ln(1.2 × (1 − x))/(1.2 − x) was plotted as a solid line.

To study the reaction profile, phenyl ester 15 was treated with benzylamine (1.2 eq.) in the presence of 20 mol% 6-chloro-2-pyridone (13a), 6-bromo-2-pyridone (13b) or 6-iodo-2-pyridone (13c) in C_6_D_6_ (1.0 M) at room temperature. The progress of this reaction was monitored by ^1^H NMR spectroscopy, and the amounts of ester 15 and product 4 were quantified to determine the conversion. The time course of conversion is plotted as a dotted line in Fig. 2. It was assumed that the reaction would proceed according to first-order kinetics to each amine (1.2 eq.) and ester (1.0 eq.). Accordingly, *kt* = (1/(1 − 1.2))ln(1.2 × (1 − *x*)/(1 × (1.2 − *x*))). In Fig. 2, time *vs.* −5 ln(1.2 × (1 − *x*))/(1.2 − *x*) (*x* = conversion, 0 ≤ *x* ≤ 1) is plotted as a solid line. As shown in this figure, 6-halo-2-pyridones 13a–c dramatically accelerated the reaction compared with catalyst-free conditions. All 2-pyridones showed similar catalytic activity, but 6-iodo-2-pyridone 13c was slightly higher. These reactions can overall be treated as a pseudo-second-order kinetics.

Next, we examined the reactions of less-reactive common esters such as methyl and benzyl esters. Methyl esters 21a, 22 and benzyl ester 21b were successfully reacted with benzylamine (1.2 eq.) in the presence of 13a (20 mol%) in toluene (1.0 M) (Table 5). Although the reaction did not proceed at ambient temperature, methyl ester 21a was consumed after stirring at 110 °C for 72 h. Corresponding amide 4 was isolated at a 99% yield through direct purification by silica gel column chromatography (entry 1). Catalyst 13a was quantitatively recovered. This reaction showed poor conversion (3%) without a catalyst. Benzyl ester 21b was also applicable, and amide 4 was obtained in 98% yield (without a catalyst, 16%; entry 2). Carboxylic acid 21c did not react at all (entry 3). Aminolysis of methyl benzoate (22) proceeded slower compared with that of aliphatic methyl ester 21a. After stirring at 110 °C for 72 h, corresponding amide 23 was obtained in 58% yield with the recovery of 22 (entry 4). It is noted that when the catalyst was changed to 2-pyridone (1), the conversion was significantly reduced to 8%.

**Table 5:** Scope and limitation, focused on esters

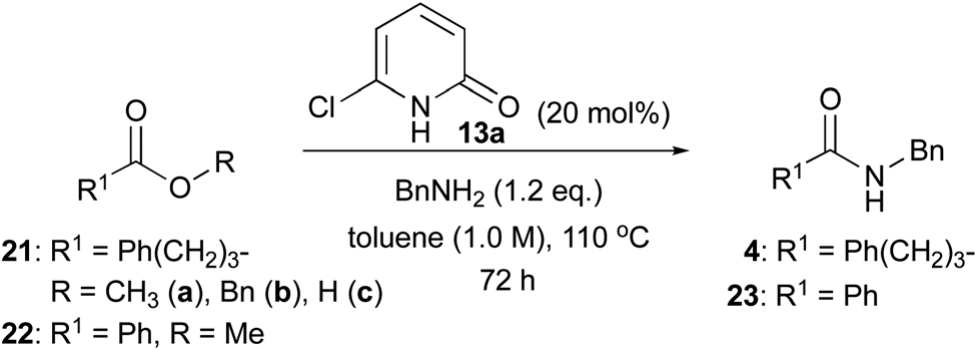				
Entry	Ester	Conversion^a^	Isolated yield	Conversion^a^ without catalyst
1	21a	>99%	99%^b^	3%
2	21b	98%	98%^b^	16%
3	21c	n.r.^c^	n.r.^c^	—
4	22	58%	58%^b^	2% (8%)^d^

a Conversion was calculated by ^1^H NMR spectroscopy.

b Catalyst was recovered quantitatively.

c No reaction.

d 2-Pyridone (1) was used as catalyst instead of 13a.

Next, we pursued the aminolysis of amino acid esters, the results of which are summarised in Table 6. Amino acid esters were found to be better substrates for this reaction compared with aliphatic acid esters such as 21a and 21b. Although the reaction was relatively slow, ester aminolysis of Boc-Gly-OBn 24a with benzylamine proceeded even at room temperature, and corresponding amide 27 was obtained in 86% isolated yield after stirring at 40 °C for 24 h (entry 1). The reaction without a catalyst proceeded at a lower conversion (34%). Unexpectedly, sterically less-hindered methyl ester 24b showed lower reactivity than 24a did (65% conversion, entry 2). We envisioned applying this reaction next to prepare a dipeptide. The treatment of l-leucine ethyl ester hydrochloride 26 with 24a in the presence of 13a (20 mol%) and NEt_3_ at 60 °C for 72 h provided dipeptide 28 in 66% isolated yield with 92% ee (entry 3). Optical purity was determined by chiral HPLC analysis. All other side products such as polypeptides, diketopiperazine and self-condensation products of 26 were not detected. This reaction did not proceed without catalyst 13a. The use of 2-pyridone (1) as a catalyst decreased the conversion to 21%. More hindered secondary amine 18 did not react even at 110 °C (entry 4). Considering the low reactivity of secondary amines, aminolysis of non-protected H-Pro-OBn 19 with benzylamine was conducted (entry 5). As anticipated, the reaction proceeded even at 40 °C to provide corresponding amide 29 in a good isolated yield (87%) while maintaining optical purity (>99% ee). In this reaction, secondary amine groups of the proline residues in substrate 19 and product 29 did not react with benzyl ester. More sterically bulky Boc-l-Leu-OBn 25 was also applicable to this aminolysis. Treatment of 25 with benzylamine at 60 °C for 94 h provided the corresponding amide 30 in 67% yield with almost maintained optical purity (99% ee, entry 6). Raising the temperature to 110 °C improved the conversion, but the optical purity decreased slightly (83% yield, 94% ee, entry 7).

**Table 6:** Aminolysis of α-aminoacetic acid ester

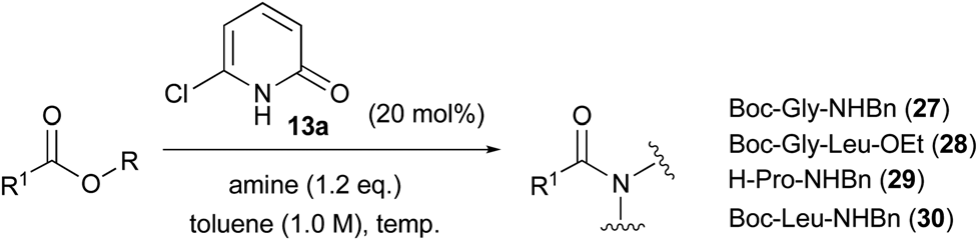								
Entry	Ester	Amine	Temp.	Time	Conversion^a^	Yield	e.e.^b^	Conversion^a^ without catalyst
1	Boc-Gly-OBn (24a)	BnNH_2_	40 °C	24 h	86%	86%	—	34%
2	Boc-Gly-OMe (24b)	BnNH_2_	40 °C	24 h	65%	n.d.	—	13%
3^c^	24a	H-l-Leu-OEt·HCl (26)	60 °C	72 h	66%	66%	92%	n.d.^d^ (21%)^e^
4^c^	24a	MeO(Me)NH·HCl (18)	110 °C	24 h	n.r.^f^	n.r.^f^	—	—
5^c^	H-l-Pro-OBn·HCl (19)	BnNH_2_	40 °C	24 h	87%	87%	>99%	11%
6	Boc-l-Leu-OBn (25)	BnNH_2_	60 °C	94 h	67%	67%	99%	—
7	25	BnNH_2_	110 °C	24 h	—	83%	94%	—

a Conversion was calculated by ^1^H NMR spectroscopy.

b Determined by chiral HPLC analysis.

c 1.2 eq. of NEt_3_ was added.

d Not detected.

e 2-Pyridone (1) was used as catalyst instead of 13a.

f No reaction.

**Fig. 3:**
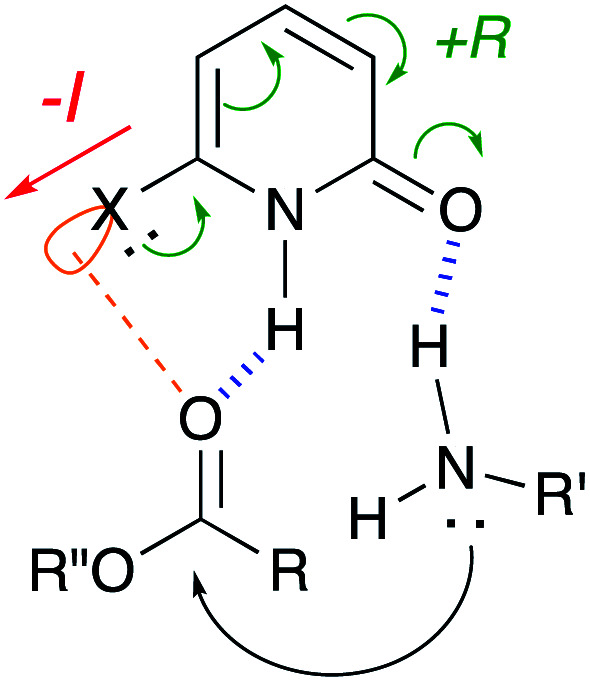
Proposed mechanism.

Based on these results, we show our proposed mechanism in Fig. 3. 2-Pyridone activates ester substrate *via* a hydrogen bond between proton of N–H in pyridone and carbonyl oxygen, and simultaneously, amine can be activated through a hydrogen bonding to pyridone carbonyl oxygen. This concurrent dual activation also makes the reaction entropically favored. 6-Halogen substitution on 2-pyridone enhances acidity of proton of N–H by negative inductive effect and basicity of carbonyl oxygen can be increased by positive resonance effect. Furthermore, it can be considered that an interaction between ester carbonyl oxygen and empty orbital of halogen atom in pyridone may participate as an additional activation. Besides these considerations, the mechanism based on a lactim tautomer, 2-hydroxypyridine (2), can not be ruled out. More detail discussion must await further study.

## Conclusions

We found that 6-halo-2-pyridones delivered remarkable catalytic activity for ester aminolysis. The catalyst enabled aminolysis of not only highly reactive aromatic esters but also ordinary methyl and benzyl esters and complete recovery of the catalyst. Biologically important amino acid derivatives apply to this reaction, which did not require any special reagents, strict dryness, anaerobic conditions or extraction and was operationally convenient. We believe that these results may enable a practical approach to catalytic peptide synthesis. Further screening of a highly reactive 2-pyridone catalyst for ester aminolysis and the development of other 2-pyridone catalysed reactions are underway in our laboratory.

## Experimental section

### General procedure for ester aminolysis

A mixture of an ester (0.1 mmol), amine (0.12 mmol) and 6-chloro-2-pyridone (20 mol%) in toluene (0.1 mL) was stirred at an appropriate temperature. The reaction was monitored by TLC. After the completion of the reaction, the mixture was put on a silica gel and purified by column chromatography to provide the corresponding amide. The catalyst 13a could be recovered quantitatively at the purification of silica gel column chromatography (hexane : AcOEt = 10 : 1).

## Conflicts of interest

There are no conflicts to declare.

## Supplementary Material

RA-011-D1RA04651A-s001
