# Supplementary material for: 6-Halo-2-pyridone as an efficient organocatalyst for ester aminolysis
Source: RSC Adv. 2021 Jul 14;11(40):24588–93. doi: 10.1039/d1ra04651a (PMC9036873; doi:10.1039/d1ra04651a)

Electronic Supplementary Material (ESI) for RSC Advances.
This journal is © The Royal Society of Chemistry 2021

6-Halo-2-pyridone as an Efficient Organocatalyst for Ester Aminolysis

Takeshi Yamada,* Yusuke Watanabe and Sentaro Okamoto*

Department of Materials and Life Chemistry, Kanagawa University, 3-27-1 Rokkakubashi, Kanagawa-ku, Yokohama, 221-8686, Japan

e-mail: tyamada@kanagawa-u.ac.jp
okamos10@kanagawa-u.ac.jp

Table of Contents

1. **General Information** **S-2**
2. **Details of Results** **S-3**
3. **Experimental Procedure and Characteristic Data** **S-6**
4. **NMR Spectra** **S-12**
5. **HPLC Spectra** **S-15**

1. General Information

All reagents and solvents were purchased from either Tokyo Chemical Industry Co., Ltd., or FUJIFILM Wako Pure Chemical Corporation, Kanto Chemical Co., Ltd., Sigma-Aldrich Co. LLC, and were used without further purification. Unless otherwise noted, all reactions were conducted without any inert gas. Chromatography was carried out with Wakogel® C-200 silica gel (FUJIFILM Wako Pure Chemical Corporation, granule, 0.075-0.150 mm). NMR spectra were recorded at 400 MHz for ^{1}H and 100 MHz for ^{13}C on JEOL JNM-ECZ400R spectrometer. Chemical shifts are reported in part per million (ppm, δ) relative to residual solvent peaks of $CDCl_3$ (7.26 ppm for ^{1}H NMR, 77.0 ppm for ^{13}C NMR) and coupling constant (*J* values) are given in Hertz. IR spectra were recorded on a JASCO IR FT/IR 4100 spectrometer. High-resolution mass spectra (HRMS) were measured on a JEOL Accu TOF T-100 equipped with an ESI ionization unit. High performance liquid chromatography was carried out with PU–2089 plus HPLC pump (JASCO), LC–NetII/ADC (JASCO), UV–2075 plus UV/Vis detector (JASCO).

2. Detail of Catalyst Screening

Table S1. Catalyst screening.

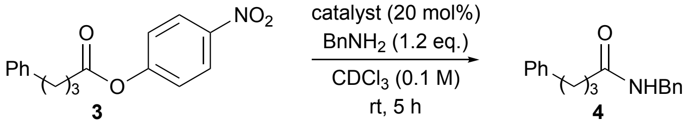

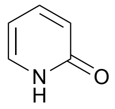	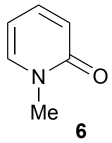	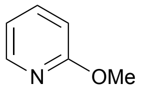	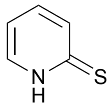	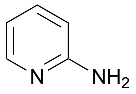	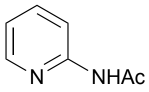
**1**	**6**	**7a**	**5**	**7b**	**7c**
37%	25%	17%	30%	20%	20%
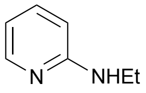	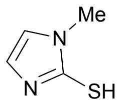	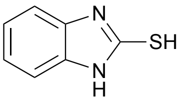	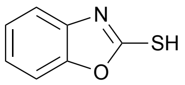	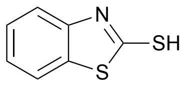	
**7d**	**8**	**9a**	**9b**	**9c**	
22%	32%	22%	30%	31%	
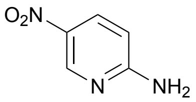	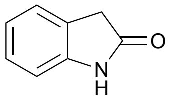	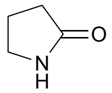	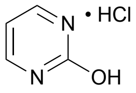	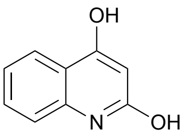	
**S1**	**S2**	**S3**	**S4**	**S5**	
20%	31%	36%	31%	16%	

Table S2. Catalyst screening.

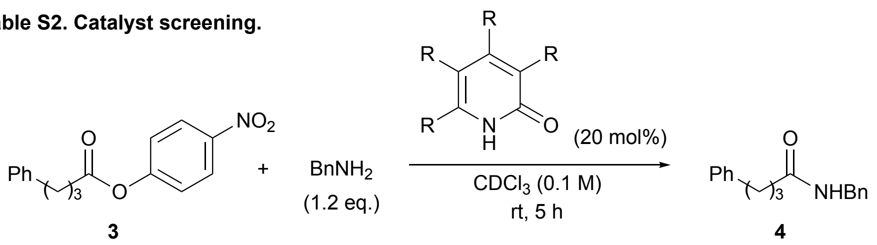

	R	Yield
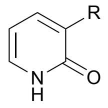	NO_2 (**10a**)	23%
	Cl (**10b**)	27%
	OMe (**10c**)	34%
	OH (**S6**)	20%
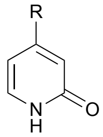	CF_3 (**11a**)	37%
	Cl (**11b**)	32%
	OMe (**11c**)	34%
	OH (**S7**)	18%
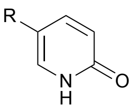	NO_2 (**12a**)	42%
	Cl (**12b**)	51%
	OMe (**12c**)	41%

	R	Yield
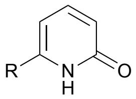	Cl (**13a**)	81%
	Br (**13b**)	78%
	I (**13c**)	86%
	F (**13d**)	31%
	CO_2Me (**13e**)	50%
	OMe (**13f**)	52%
	CH_3 (**13g**)	34%
	CO_2H (**S8**)	16%
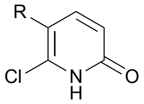	Cl (**14a**)	58%
	NO_2 (**14b**)	22%
	CO_2H (**S9**)	6%
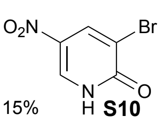 15% **S10**	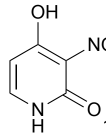 **S11** 15%	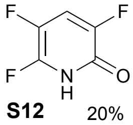 **S12** 20%

Table S3. Study of solvent effect.

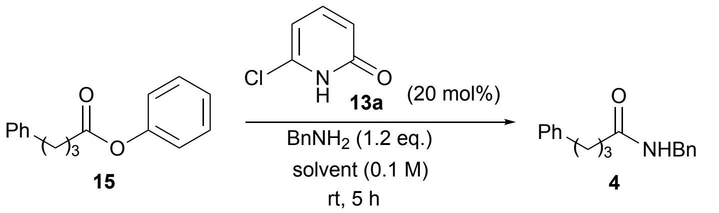

Entry	solvent	Conversion[a]	Conversion[a] without catalyst
1	$CHCl_3$	24%	-
2	CH_2Cl_2	34%	<1%
3	THF	18%	-
4	Et_2O	29%	2%
5	DME	22%	-
6	Benzene	46%	-
7	Toluene	52%	2%
8	Chlorobenzene	48%	-
9	Trifluoromethylbenzene	48%	2%
10	Hexane	74%	3%
11	DMF	13%	-
12	DMSO	30%	-
13	MeCN	41%	<1%
14	Pyridine	10%	-
15	*t*-BuOH	18%	-

[a] Conversion was calculated based on ^{1}H NMR spectroscopy.

3. Experimental Procedure and Characteristic Data

Catalyst **7c**,[1] **7d**,[2] **10c**,[3] **11c**,[4] **13e**,[5] **13f**,[6] **14a**,[7] **14b**[8] and ester **21a**,[9,10] were prepared by using known methods. Experimental procedures for preparing catalyst **12c, 13c** and esters **3**, **15, 21b, 24a, 25** are described below. Other catalysts and substrates were purchased from commercial suppliers.

Preparation of 5-methoxy-2-pyridone (12c)

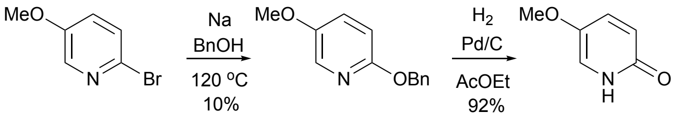

12c

To a stirred BnOH (12 mL) was added Na (304 mg, 13.2 mmol) at 0 °C under Ar atmosphere. The reaction mixture was warmed to 100 °C, then 2-bromo-5-methoxypyridine (2.26 g, 12.0 mmol) was added to the mixture. After stirring the mixture at 120 °C for 24 h, the reaction mixture was cool to rt, then the mixture was put on a silica gel and purified by column chromatography to give a 2-(benzyloxy)-5-methoxypyridine (251 mg, 10%). To a stirred solution of 2-(benzyloxy)-5-methoxypyridine (251 mg, 1.2 mmol) in AcOEt (6 mL) was added 10 wt% Pd/C (25 mg) under Ar atmosphere. Ar was replaced by H_2, then the mixture was stirred at room temperature for 30 min. The suspension was filtered through a pad of celite® and the filtrate was concentrated *in vacuo*. to provide a 5-methoxy-2-pyridone (**12c**) (135 mg, 92%). The characteristic data was consistent with reported value.[11]

Preparation of 6-iodo-2-pyridone (13c)[12]

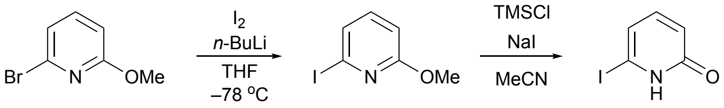

13c

---

[1] J. Zeng, Y. J. Tan, M. L. Leow, X.-W. Liu, *Org. Lett.* 2012, **14**, 4386-4389.
[2] O. M. Singh, S. J. Singh, S. N. Kim, S.-G. Lee, *Bull. Korean Chem. Soc.* 2007, **28**, 115-117.
[3] S. Hanessian, O. M. Saavedra, V. Mascitti, W. Marterer, R. Oehrlein, C.-P. Mak, *Tetrahedron* 2001, **16**, 3267-3280.
[4] S. McN. Sieburth, C.-H. Lin, D. Rucando, *J. Org. Chem.* 1999, **64**, 950-953.
[5] H.-B. Zhou, G.-S. Liu, Z.-J. Yao, *Org. Lett.* 2007, **9**, 2003-2006.
[6] C. Kaneko, K. Uchiyama, M. Sato, N. Katagiri, *Chem. Pharm. Bull.* 1986, **34**, 3658-3671.
[7] M. J. Bingham, M. J. Huggett, M. Huggett, Y. Kiyoi, S. E. Napier, O. Nimz, *PCT Int. Appl.*, 2007039563 A1.
[8] A. T. Plowright, P. Barton, S. Bennett, A. M. Birch, S. Birtles, *et al.*, *Med. Chem. Commun.* 2013, **4**, 151-158.
[9] Y. Baek, T. A. Betley, *J. Am. Chem. Soc.* 2019, **141**, 7797-7806.
[10] T. O. Vieira, M. J. Green, H. Alper, *Org. Lett.* 2006, **8**, 6143-6145.
[11] V. S. Chan, S. W. Krabbe, C. Li, L. Sun, Y. Liu, A. J. Nett, *ChemCatChem*, 2019, **11**, 5748-5753.
[12] Y. Yuan, W. Dong, X. Gao, H. Gao, X. Xie, Z. Zhang, *J. Org. Chem.* 2018, **83**, 2840-2846.

To a stirred solution of 2-bromo-6-methoxypyridine (0.86 mL, 7.1 mmol) in THF (11 mL) was added n-BuLi (1.56 M in hexane, 5 mL, 7.8 mmol) at –78°C under Ar atmosphere. The reaction mixture was stirred at same temperature for 1 h, then a solution of I_2 (2.16 g, 8.5 mmol) in THF (10 mL) was added. The reaction mixture was gently warm to room temperature. The reaction was quenched with a saturated aqueous NH_4Cl (40 mL) and extracted by AcOEt (30 mL x 3). Combined organic layers were washed with brine (40 mL) and dried over anhydrous Na_2SO_4. The mixture was filtered and concentrated *in vacuo*. To a stirred suspension of crude mixture and NaI (3.2 g, 21 mmol) in MeCN (36 mL) was added TMSCl (4.5 mL, 35.6 mmol) at room temperature under Ar atmosphere. After the reaction mixture was stirred at same temperature for 13 h, the reaction was quenched with a saturated aqueous NaS_2O_3 (40 mL) and extracted by CH_2Cl_2 (30 mL x 3). Combined organic layers were washed with brine (40 mL) and dried over anhydrous Na_2SO_4. The mixture was filtered and concentrated *in vacuo*. A Et2O (5 mL) was added the residue and the insoluble material was filtered off and washed with Et2O (5 mL x 2). The combined Et2O solution was concentrated *in vacuo*. and the residue was purified by flash column chromatography on silica gel (Hexane : AcOEt = 10 : 1) to give a 6-iodo-2-pyridone (**13c**) (360 mg, 23%) as a colorless solid and 2-iodo-6-methoxypyridine (809 mg, 49%) as a colorless oil.

Characteristic data of 6-iodo-2-pyridone (**13c**)

^{1}H NMR (400 MHz, $CDCl_3$): δ 6.63 (dd, J = 9.0, 0.8 Hz, 1H), 6.80 (dd, J = 7.0, 0.8 Hz, 1H), 7.15 (dd, J = 9.0, 7.0 Hz, 1H). ^{13}C NMR (100 MHz, $CDCl_3$): δ 97.3, 117.6, 120.0, 141.6, 165.6. IR (neat, ATR) 2837, 2360, 2338, 1633, 1574, 1533, 1450, 1160, 977, 914, 789 cm^{-1}. HRMS (ESI-TOF) m/z $[M + Na]^+$ calcd for $[C_5H_5INO_4]^+$ 221.9416, found 221.9413.

Preparation of 4-phenylbutanoic acid *p*-nitrophenylester (3)

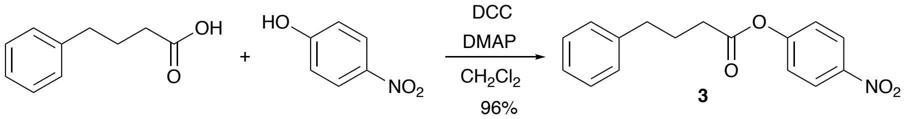

To a stirred solution of 4-nitrophenol (556 mg, 4.0 mmol) and 4-phenylbutanoic acid (657 mg, 4.0 mmol) in CH_2Cl_2 (40 mL) was added DCC (908 mg, 4.4 mmol) and DMAP (49 mg, 0.4 mmol) at 0 °C under Ar atmosphere. After stirring the mixture at room temperature for 13 h, the reaction was quenched with a saturated aqueous NH_4Cl (30 mL) and extracted by AcOEt (30 mL x 3). Combined organic layers were washed with brine (30 mL) and dried over anhydrous Na_2SO_4. The mixture was filtered and concentrated *in vacuo*. The residue was purified by flash column chromatography on silica gel (Hexane : AcOEt = 20 : 1) to give a 4-nitrophenyl ester **3** (1.10 g, 96%) as a colorless solid.

^{1}H NMR (400 MHz, $CDCl_3$): δ 2.11 (tt, J = 7.4, 7.4 Hz, 2H), 2.62 (t, J = 7.4 Hz, 2H), 2.76 (t, J = 7.4 Hz, 2H), 7.19-7.28 (5H), 7.32 (m, 2H), 8.27 (m, 2H). ^{13}C NMR (100 MHz, $CDCl_3$): δ 26.2, 35.5, 34.9, 122.4 (2C), 125.2 (2C), 126.2, 128.5 (4C), 140.8, 145.2, 155.4, 171.0. IR (neat, ATR) 2933, 1762, 1591, 1523, 1491, 1345,

1204, 1161, 1114, 920, 861, 746 cm^{-1}. HRMS (ESI-TOF) m/z $[M + Na]^+$ calcd for $[C_{16}H_{15}NNaO_4]^+$ 308.0899, found 308.0890.

Preparation of 4-phenylbutanoic acid phenylester (15)

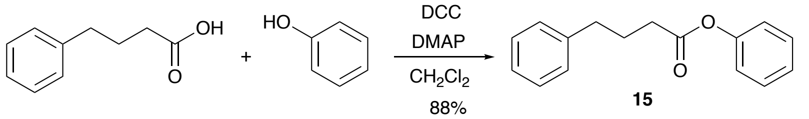

To a stirred solution of phenol (564 mg, 6.0 mmol) and 4-phenylbutanoic acid (985 mg, 6.0 mmol) in CH_2Cl_2 (60 mL) was added DCC (1.36 g, 6.6 mmol) and DMAP (73.3 mg, 0.6 mmol) at 0 ºC under Ar atmosphere. After stirring the mixture at room temperature for 13 h, the reaction was quenched with a saturated aqueous NH_4Cl (40 mL) and extracted by AcOEt (30 mL x 3). Combined organic layers were washed with brine (50 mL) and dried over anhydrous Na_2SO_4. The mixture was filtered and concentrated *in vacuo*. The residue was purified by flash column chromatography on silica gel (Hexane : AcOEt = 20 : 1) to give a phenyl ester **15** (1.27 g, 88%) as a colorless solid. The characteristic data were consistent with reported value.[13]

Preparation of benzylester (21b)

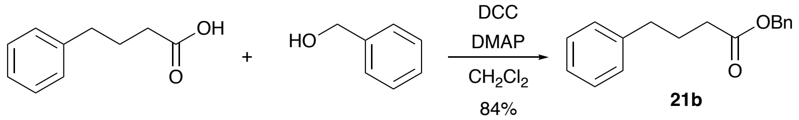

To a stirred solution of benzyl alcohol (0.62 mL, 6.0 mmol) and 4-phenylbutanoic acid (985 mg, 6.0 mmol) in CH_2Cl_2 (60 mL) was added DCC (1.36 g, 6.6 mmol) and DMAP (73.3 mg, 0.6 mmol) at 0 ºC under Ar atmosphere. After stirring the mixture at room temperature for 13 h, the reaction was quenched with a saturated aqueous NH_4Cl (40 mL) and extracted by AcOEt (30 mL x 3). Combined organic layers were washed with brine (50 mL) and dried over anhydrous Na_2SO_4. The mixture was filtered and concentrated *in vacuo*. The residue was purified by flash column chromatography on silica gel (Hexane : AcOEt = 20 : 1) to give a phenyl ester **2ab** (1.27 g, 84%) as a colorless solid. The characteristic data were consistent with reported value.[14]

Preparation of Boc-Gly-OBn (24a)

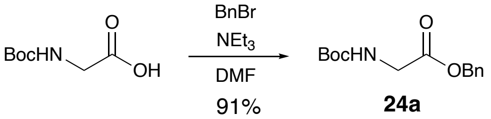

To a stirred solution of Boc-Gly-OH (1.75 g, 10.0 mmol) and NEt_3 (1.7 mL, 12.0 mmol) in DMF (40

---

[13] M. S. Carle, G. K. Shimokura, G. K. Murphy, *Eur. J. Org. Chem.*, 2016, 3930-3933.

[14] W. Wang, H. Liu, S. Xu, Y. Gao, *Syn. Commun.*, 2013, **43**, 2906-2912.

mL) was added benzyl bromide (2.4 mL, 20 mmol) at 0 ºC under Ar atmosphere. After stirring the mixture at room temperature for 17 h, the reaction was quenched with a saturated aqueous NH_4Cl (30 mL) and extracted by Et_2O (20 mL x 3). Combined organic layers were washed with brine (30 mL) and dried over anhydrous Na_2SO_4. The mixture was filtered and concentrated *in vacuo*. The residue was purified by flash column chromatography on silica gel (Hexane : AcOEt = 9 : 1) to give a benzyl ester **24a** (2.41 g, 91%) as a colorless oil. The characteristic data were consistent with reported value.[15]

Preparation of Boc-L-Leu-OBn (25)

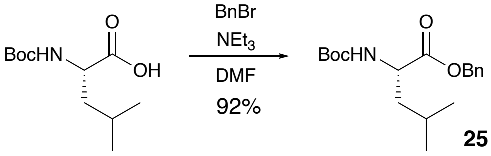

To a stirred solution of Boc-Leu-OH (2.31 g, 10.0 mmol) and NEt_3 (1.7 mL, 12.0 mmol) in DMF (40 mL) was added benzyl bromide (2.4 mL, 20 mmol) at 0 ºC under Ar atmosphere. After stirring the mixture at room temperature for 17 h, the reaction was quenched with a saturated aqueous NH_4Cl (30 mL) and extracted by Et_2O (20 mL x 3). Combined organic layers were washed with brine (30 mL) and dried over anhydrous Na_2SO_4. The mixture was filtered and concentrated *in vacuo*. The residue was purified by flash column chromatography on silica gel (Hexane : AcOEt = 15 : 1) to give a benzyl ester **25** (2.96 g, 92%) as a colorless oil. The characteristic data were consistent with reported value.[16]

General Procedure for Ester Aminolysis catalyzed by 2-Pyridone

A mixture of an ester (0.1 mmol), amine (0.12 mmol) and 6-chloro-2-pyridone **13a** (20 mol%) in toluene (0.1 mL) was stirred. The reaction was monitored by TLC. After completion of the reaction, the mixture was put on silica gel and purified by column chromatography to give the corresponding amide. The catalyst **13a** could be recovered quantitatively at the purification of silica gel column chromatography (hexane : AcOEt = 10 : 1).

---

[15] C. Wiles, P. Watts, S. J. Haswell, E. Pombo-Villar, *Tetrahedron* 2003, **59**, 10173-10179.

[16] F. E. Dutton, B. H. Lee, S. S. Johnson, E. M. Coscarelli, P. H. Lee, *J. Med. Chem.* 2003, **46**, 2057-2073.

Characteristic data of amides **4**,[17] **S13**,[18] **S14**,[19] **S15**,[20] **23**,[21] **27**,[22] **28**,[23] **29**,[24] **30**[25] were consistent with reported value.

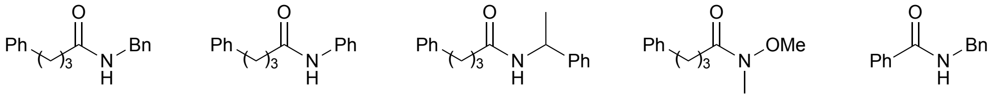

4 **S13** **S14** **S15** **23**

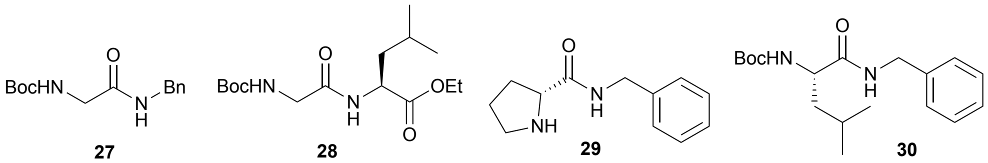

27 **28** **29** **30**

4-Phenylbutanoic acid prolinamide (S16)

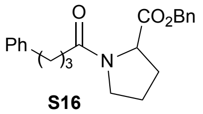

S16

Rotamer was detected in ^{1}H and ^{13}C NMR spectra. (1 : 0.2)

^{1}H NMR (400 MHz, $CDCl_3$): δ 1.81-2.39 (9.6 H), 2.56 (m, 0.4 H), 2.67 (t, J =7.4 Hz, 2H), 3.40 (m, 1H), 3.50-3.59 (1.2 H), 3.64 (ddd, J = 5.9, 5.9, 5.9 Hz, 0.2 H), 4.30 (dd, J = 8.4, 2.4 Hz, 2H), 4.54 (dd, J = 8.6, 3.4 Hz, 2H), 5.12 (s, 0.4 H), 5.12 (d, J = 12.4 Hz, 1H), 5.20 (d, J = 12.4 Hz, 1H), 7.10-7.40 (12.3 H). ^{13}C NMR (100 MHz, $CDCl_3$): 22.5, 24.7, 26.0, 26.1, 29.1, 31.4, 33.4, 33.4, 35.0, 35.1, 46.3, 46.9, 58.7, 59.4, 66.7, 67.1, 125.8, 128.0 (2C), 128.1, 128.2 (2C), 128.3, 128.4, 128.5 (2C), 128.5 (2C), 128.6, 128.6, 135.2, 135.7, 141.7, 171.6, 172.0, 172.2. IR (neat, ATR) 2949, 2361, 1740, 1643, 1421, 1166, 1024, 743 cm^{-1}. HRMS (ESI-TOF) m/z [M + Na]+ calcd for $[C_{22}H_{25}NNaO_3]^+$ 374.1732, found 374.1728.

Boc-Gly-L-Leu-OEt (28)[23]

---

[17] T. Maki, K. Ishihara, H. Yamamoto, *Org. Lett.* 2005, **7**, 5043-5046.

[18] K. Ishihara, S. Ohara, H. Yamamoto, *J. Org. Chem.*, 1996, **61**, 4196-4197.

[19] K. Arnold, B. Davies, D. Hérault, A. Whitting, *Angew. Chem. Int. Ed.* 2008, **47**, 2673-2676.

[20] T. E. La Cruz, S. D. Rychnovsky, *Chem. Commun.*, 2004, 168-169.

[21] T. Maki, K. Ishihara, H. Yamamoto, *Org. Lett.* 2006, **8**, 1431-1434

[22] J. C. Barrow, K. E. Rittle, P. L. Ngo, H. G. Selnick, S. L. Graham, *et al.*, *ChemMedChem* 2007, **2**, 995-999.

[23] D. N. Sawant, D. B. Bagal, S. Ogawa, K. Selvam, S. Saito, *Org. Lett.* 2018, **20**, 4397-4400.

[24] I. Held, E. Larionov, C. Bozler, F. Wagner, H. Zipse, *Synthesis*, 2009, **13**, 2267-2277.

[25] K. C. Nadimpally, K. Thalluri, N. B. Palakurthy, A. Saha, B. Mandal, *Tetrahedron Lett.* 2011, **52**, 2579-2582.

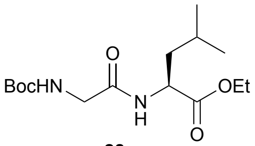

28

CHIRAL-Cel OD-H, *i*-PrOH : Hexane = 4 : 96, UV 254 nm, flow rate 1.0 mL/min, L-isomer t_R = 6.78 min, D isomer t_R = 6.04 min. 92.1% ee.

L-Proline benzylamide (29)[24]

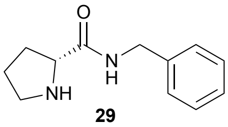

29

CHIRAL-PAK IB N-5, *i*-PrOH (0.1% Ethanolamine) : Hexane (0.1% Ethanolamine) = 1 : 4, UV 254 nm, flow rate 1.0 mL/min, L-isomer t_R = 6.917 min, D isomer t_R = 7.38 min. >99% ee.

Boc-L-Leu-NHBn (30)[25]

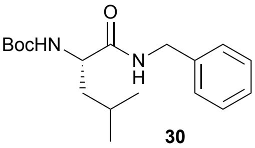

30

CHIRAL-Cel OD-H, *i*-PrOH : Hexane = 3 : 97, UV 243 nm, flow rate 1.0 mL/min, L-isomer t_R = 10.56 min, D isomer t_R = 7.67 min.

^1H NMR (400 MHz, $CDCl_3$) spectra of 6-iodo-2-pyridone **13c**

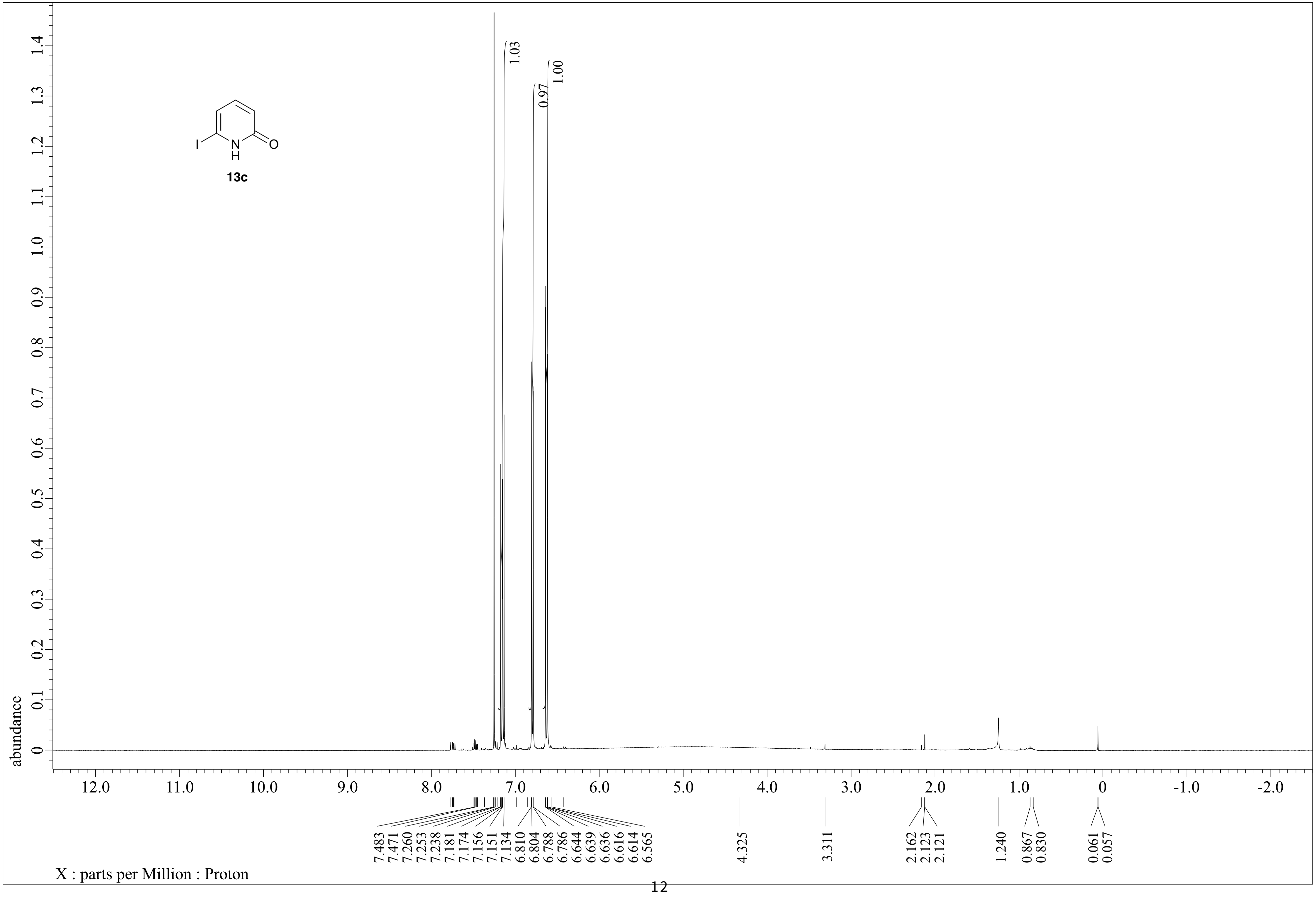

^{1}H NMR (400 MHz, $CDCl_3$) spectra of *p*-nitrophenylester **3**

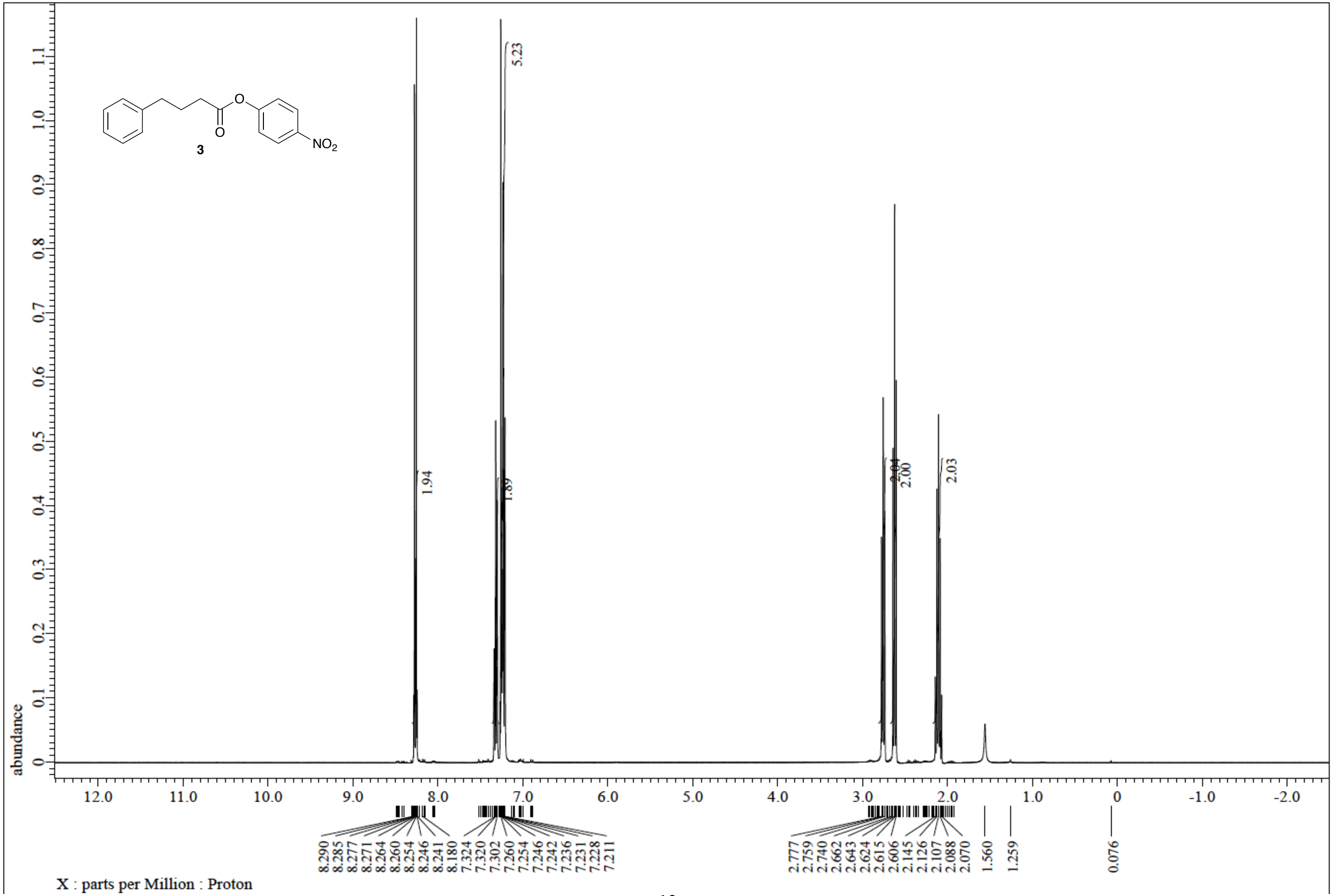

^{1}H NMR (400 MHz, $CDCl_3$) spectra of **S16**

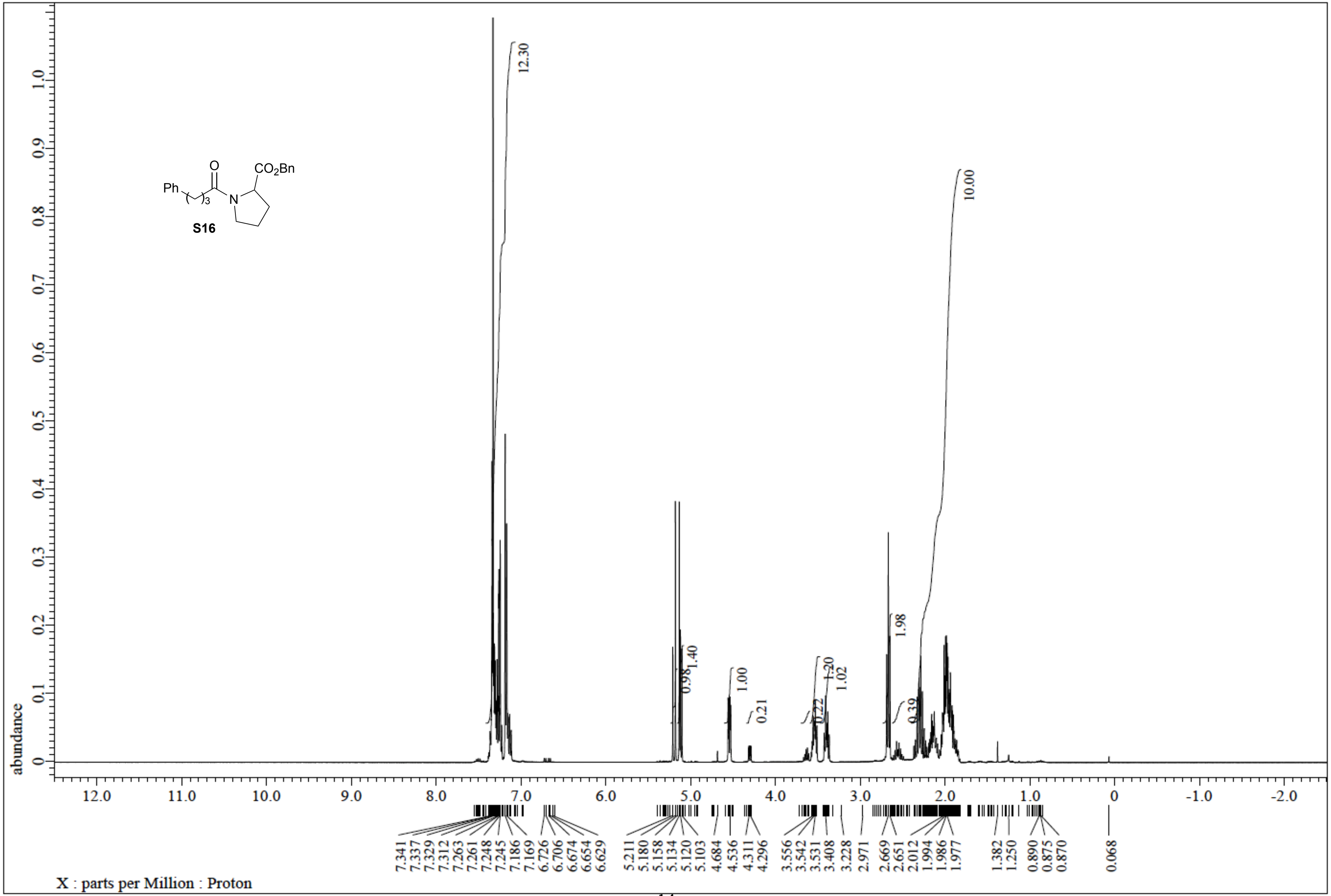

Chiral HPLC analysis of Boc-Gly-L-Leu-OEt (**28**)

CHIRAL-Cel OD-H, *i*-PrOH : Hexane = 4 : 96, UV 254 nm, flow rate 1.0 mL/min, L-isomer t_R = 6.78 min, D isomer t_R = 6.04 min. 92.1% ee.

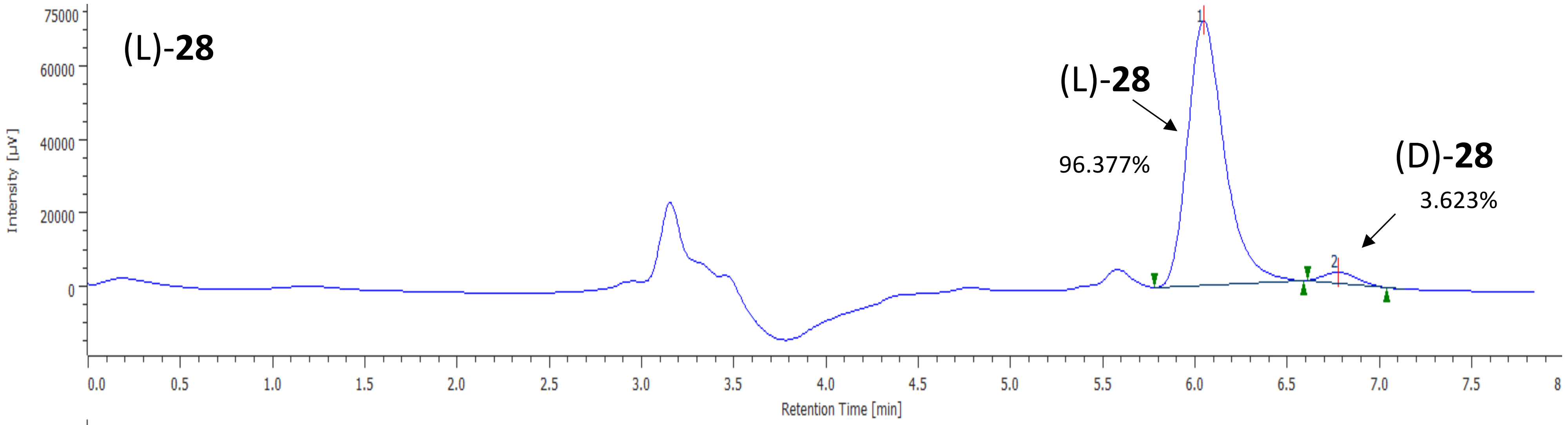

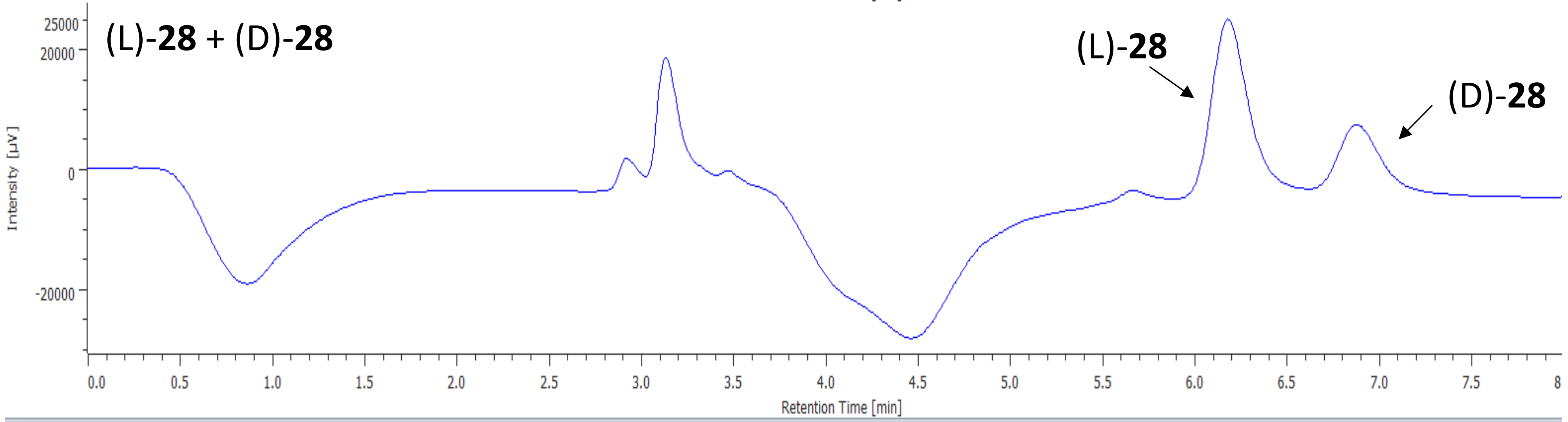

ピーク情報: ty-20-10-7-PrNHBn

#	ピーク名	CH	tR	面積	高さ	面積%	高さ%	定量値	NTP	分離度	シンメトリー係数	警告
1	Unknown	1	6.042	999255	72031	96.377	96.049	N/A	4719	2.092	1.375	
2	Unknown	1	6.775	37569	2963	3.623	3.951	N/A	5959	N/A	1.232	

Chiral HPLC analysis of L-Proline benzylamide (**29**)

CHIRAL-PAK IB N-5, *i*-PrOH (0.1% Ethanolamine) : Hexane (0.1% Ethanolamine) = 1 : 4, UV 254 nm, flow rate 1.0 mL/min, L-isomer t_R = 6.92 min, D isomer t_R = 7.38 min. >99% ee.

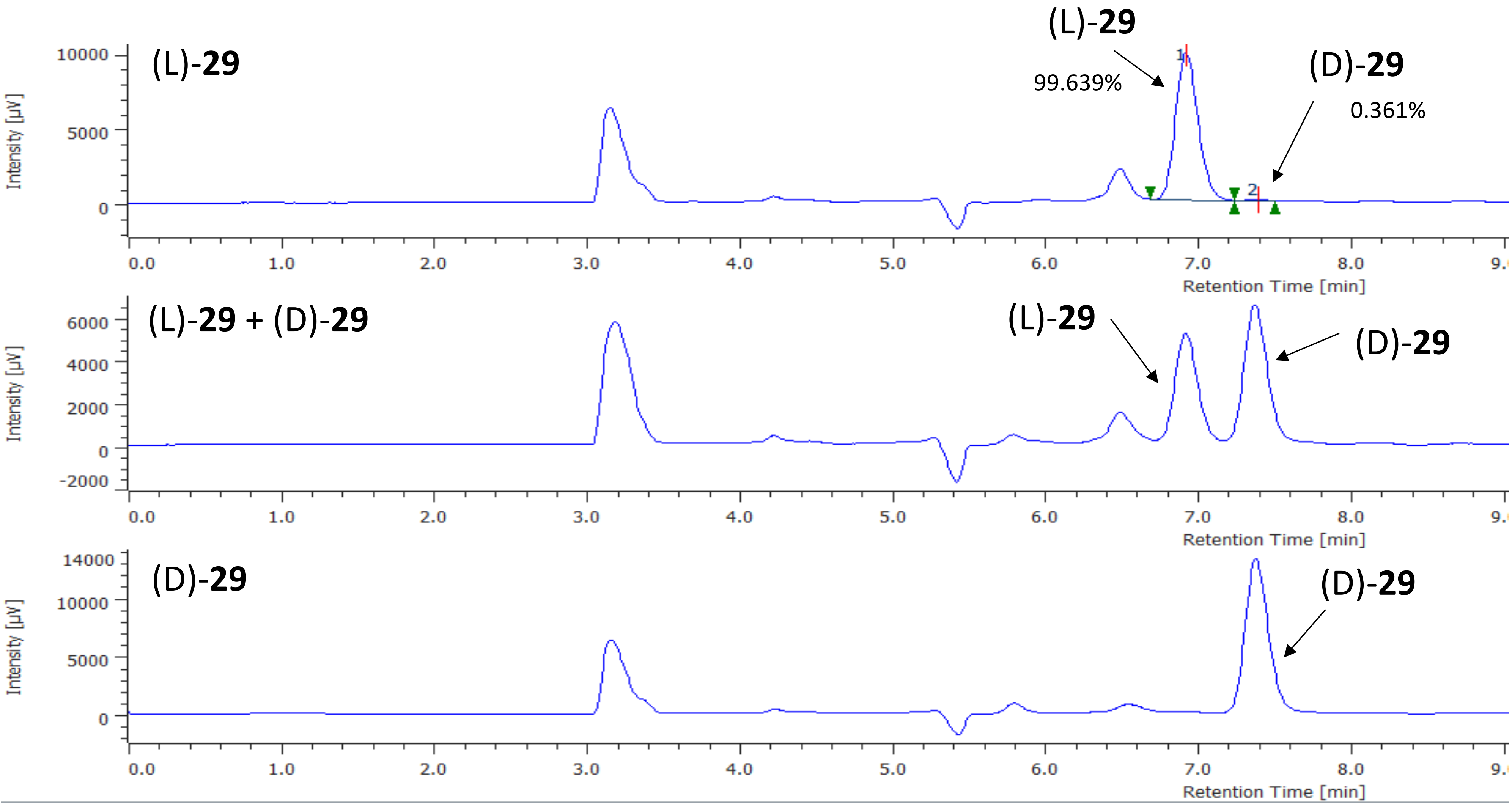

ピーク情報: aminolysi

#	ピーク名	CH	tR	面積	高さ	面積%	高さ%	定量値	NTP	分離度	シンメトリー係数	警告
1	Unknown	1	6.917	99157	9713	99.639	99.513	N/A	10536	1.871	1.137	
2	Unknown	1	7.383	360	48	0.361	0.487	N/A	16404	N/A	0.992	

Chiral HPLC analysis of Boc-L-Leu-NHBn (**30**)

CHIRAL-Cel OD-H, *i*-PrOH : Hexane = 3 : 97, UV 243 nm, flow rate 1.0 mL/min, L-isomer t_R = 10.56 min, D isomer t_R = 7.67 min.

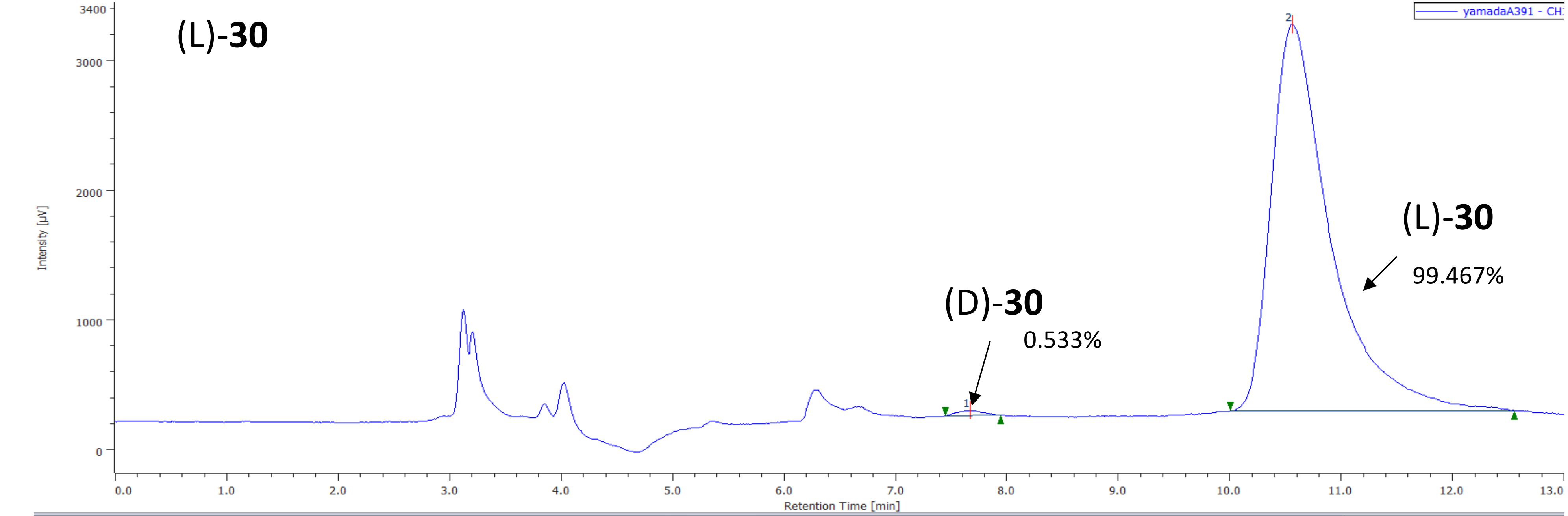

ピーク情報: yamadaA391

#	ピーク名	CH	tR	面積	高さ	面積%	高さ%	定量値	NTP	分離度	シンメトリー係数	警告
1	Unknown	1	7.667	589	40	0.533	1.320	N/A	4447	4.345	1.041	
2	Unknown	1	10.558	109951	2979	99.467	98.680	N/A	2331	N/A	1.875	

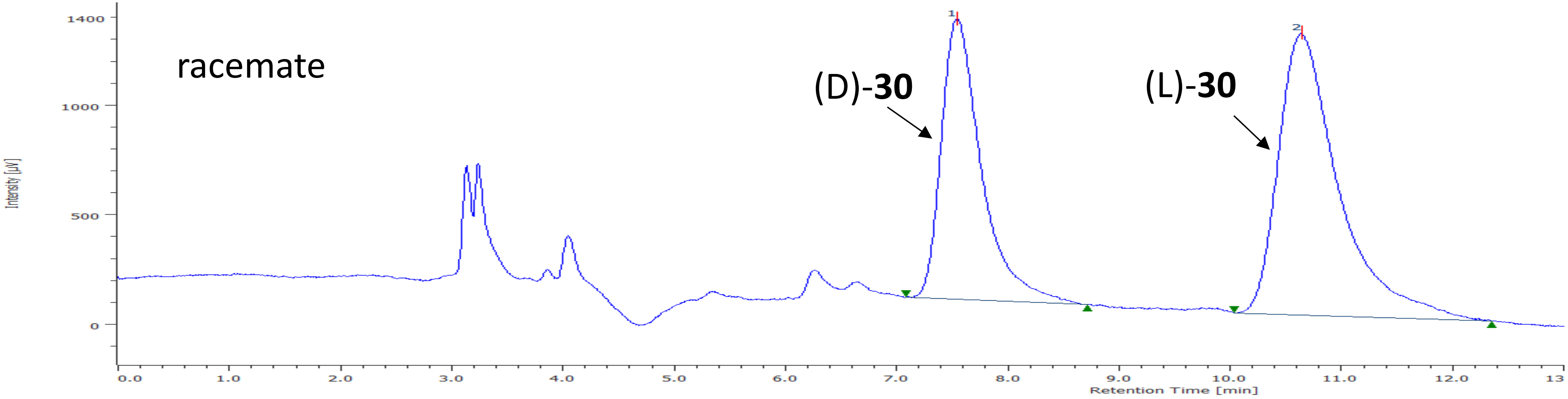

Supplement: RA-011-D1RA04651A-s001 [file RA-011-D1RA04651A-s001.pdf]
